# Editorial: Prokaryotic Communications: From Macromolecular Interdomain to Intercellular Talks (Recognition) and Beyond

**DOI:** 10.3389/fmolb.2021.670572

**Published:** 2021-04-21

**Authors:** Chew Chieng Yeo, Manuel Espinosa, Tatiana Venkova

**Affiliations:** ^1^Centre for Research in Infectious Diseases and Biotechnology (CeRIDB), Faculty of Medicine, Universiti Sultan Zainal Abidin, Kuala Terengganu, Malaysia; ^2^Margarita Salas Center for Biological Research, Spanish National Research Council, Madrid, Spain; ^3^Fox Chase Cancer Center, Philadelphia, PA, United States

**Keywords:** prokaryotic communications, quorum sensing, intermolecular communication, intramolecular communication, molecular recognition

## Introduction

Communication is integral to all life on Earth. From complex microbial communities to the regulation of fundamental cellular processes such as DNA replication and transcription, intra- and inter-molecular communications, macromolecular cross-talks, and cell-cell communication lies at the heart of these processes. The concept of intercellular communication in the prokaryotic world came from discoveries made around 50 years ago when Tomasz reported that competence for natural transformation in *Streptococcus pneumoniae* requires a “hormone-like activator” that synchronize the bacterial population for entry into the competent state (Tomasz, 1965). Five years later in 1970, it was reported that the bioluminescent marine bacteria, *Vibrio fischeri* and *Vibrio harveyi* only produced light at high cell density but not when the bacterial cultures were diluted (Nealson et al., 1970). It took another 10 years or so before acyl-homoserine lactone (AHL) was identified as the component, termed the autoinducer, that was responsible for the stimulation of light production in the vibrios (Eberhard et al., 1981) and another 10–15 years before the concept of quorum sensing was firmly established along with its ever-growing lexicon of chemical languages as outlined in the seminal review by (Bassler and Losick, 2006). Besides quorum sensing, other forms of intercellular communications play important roles in microbial interactions. These include secretion systems such as the type VI secretion system (T6SS) that is central to predator-prey interactions (Cherrak et al., 2019), extracellular or membrane vesicles that transfer a multitude of biomolecules including DNA and RNA (Bose et al., 2021) and appendages such as conjugative pili that mediates the horizontal transfer of DNA through conjugation (Virolle et al., 2020). This Research Topic not only covers intercellular communication among bacteria but also the gamut of processes that govern intracellular communication within each bacterial cell.

## Overview of Manuscripts in this Research Topic

T6SS is a macromolecular multiprotein complex that functions to deliver killer toxins from the donor bacterium (predator) into the cytoplasm of the target cell (prey) which can either be another bacterium or a eukaryotic cell. Most T6SS are dependent on cell-cell contact and, in some cases, are regulated by quorum sensing networks. T6SS are also influenced by bacterial conjugation and associated genetic elements including plasmids and integrative and conjugative elements (ICEs) and this is the subject of a mini-review by Peñil-Celis and Garcillán-Barcia.

Conjugation is one of the most widespread mechanisms of horizontal gene transfer in bacteria (Virolle et al., 2020) and one of its consequences is the widespread dissemination of antimicrobial resistance genes. The World Health Organization has recognized antibiotic resistance as one of the major threats to public health (Tacconelli et al., 2018). Due to the vital nature of type IV coupling proteins (T4CPs) in the conjugative process, they have become appealing targets in the quest to inhibit bacterial conjugation and, in parallel, to limit the spread of antimicrobial resistance. Two papers were contributed by Itziar Alkorta's research group on T4CPs: Álvarez-Rodríguez, Ugarte-Uribe et al. studied the functionality of T4CPs, especially its transmembrane domain (TMD), in plasmid transfer, their secondary structure, thermal stability, and subcellular localization. Álvarez-Rodríguez, Arana et al. also reviewed our current state of knowledge on T4CPs and explore the possibility of using T4CP inhibitors to impede the spread of antimicrobial resistance.

The IncH1 conjugative plasmids, of which the R27 plasmid is its prototype, have been associated with multidrug resistance in pathogens such as *Salmonella enterica* and *Escherichia coli* (Holt et al., 2011). An interesting feature of the IncH1 plasmids is the temperature-dependence of their conjugation whereby higher conjugative frequencies were observed at low temperatures of between 22 and 30°C. Gibert et al. utilized a transcriptomics approach to characterize the effect of temperature on the expression of R27-encoded genes leading to the finding that the HtdA-TrhR/TrhY regulatory circuit is a likely mediator for the environmental regulation of R27 gene expression. The different regulatory circuits involved in the control of conjugation in the *Bacillus subtilis* plasmid pLS20 are reviewed by Meijer et al. who focuses on the proteins that control the activity of the main promoter, Pc, located upstream of the conjugation operon.

Two integral membrane thermosensor histidine kinases from the Gram-positive pathogens *Staphylococcus aureus* and *Bacillus anthracis* were identified by Fernández et al. who also showed that these histidine kinases likely control the expression of putative ATP-binding cassette (ABC) transporters and were regulated by environmental temperature.

Another Gram-positive pathogen, *Streptococcus pneumoniae*, is well-known to develop competence for the uptake of DNA by natural transformation and this ability enables the pneumococci to have highly diverse genomes, making them well-equipped for adaptation (Andam and Hanage, 2015). SigX (σ^X^) is the only known alternative σ-factor in streptococci and is the master competence regulator that enables transcription from competence-specific combox promoters. However, the pneumococcal transformation also requires ComW and Innis and Morrison show that a DNA-binding ComW variant, ComWΔ6, can stimulate transcription from σ^X^ promoters *in vitro*.

In another paper on *S. pneumoniae*, Li et al. demonstrated that the PsrA tyrosine recombinase controls DNA inversions that are mediated by three inverted repeats in three DNA methyltransferase *hsdS* genes of the type I restriction-modification *cod* locus. This leads to diversification of genomic DNA methylation patterns and phenotypic phase variation in colony opacity, which in turn, leads to adaptation of the pneumococci in colonization and virulence. Interestingly, PsrA activity relies on Mg^2+^ ions, unlike other site-specific tyrosine recombinases.

Gene duplications are characteristic of bacterial genomes and it is another way of generating functional diversity and increasing genomic complexity in prokaryotes. Sanchez-Herrero et al. analyzed the extent of gene duplications in the genomes of three pathogens, *Staphylococcus aureus, Enterococcus faecium* and *Enterococcus faecalis*, and found an irregular distribution of duplications in the genomes of the analyzed strains. Although mobile DNA accounted for the majority of the duplicated genes, duplication of core genes was also discovered.

The mechanism of segregation of the low copy number multidrug resistance plasmid TP228 in *Escherichia coli* was explored by Caccamo et al. who presented a unique “Venus flytrap” mechanism involving interactions between the ParF ATPase and the ParG centromere-binding protein. Replication of the Shiga toxin-converting bacteriophages (Stx phages), which are responsible for the virulence of enterohemorrhagic *E. coli* strains, was investigated by Kozłowska et al. An essential step in replication initiation is the binding of the phage-encoded O protein to iterons in the *origin* of replication region of which Stx phages has six as compared to four iterons in phage λ. Kozłowska et al. described the binding of the Stx-encoded O protein to their cognate iterons and compared them to those that occur during the formation of replication complexes in phage λ. The effects of pH on the activity of the Gram-positive plasmid pMV158-encoded RepB replication initiation protein was examined by Valdelvira et al. who showed that despite acidic pH impairing the endonuclease activity of RepB *in vitro*, the ability of pMV158 to replicate in *Lactococcus lactis in vivo* was largely unaffected at extracellular pHs that ranged from 5.0 to 7.0.

The important roles of small regulatory RNAs (sRNAs) and small proteins in intercellular communication and the control of various cellular processes have only been elucidated recently (Wagner and Romby, 2015). Ul Haq et al. reviews our current knowledge of interactions involving sRNA-mRNA, sRNA-protein and small protein-protein interactions in the Gram-positive *Bacillus subtilis*. In another article on the molecular interactions in *B. subtilis*, Moreno-del Alamo et al. showed that the PcrA protein, which is crucial in cell viability, functions at the interface of DNA replication, transcription, recombination, and segregation by working in tandem with recombination and repair proteins at stalled DNA polymerase/RNA polymerase complexes to facilitate replication beyond any conflict points.

Jeon et al. investigated the RNase E-mediated decay of polycistronic mRNA in *E. coli*, a major mechanism for the regulation of gene expression. A global analysis was performed by Huertas-Rosales et al. to identify RNA sequences bound *in vivo* by three post-transcriptional regulators of the RsmA/CsrA family in *Pseudomonas putida* KT2440 leading to an overview of the network of genes and processes subjected to post-transcriptional regulation in a bacterium that harbors three Rsm homologs.

Hernando-Amado et al. investigated the molecular basis of the plant flavonoid naringenin as an anti-quorum sensing compound and showed that naringenin directly binds to the quorum-sensing regulator LasR of *Pseudomonas aeruginosa*.

The role of the bacterial cytoskeleton in cell-cell communication along with cellular apparatuses that function in contact-dependent signaling is extensively reviewed by Singhi and Srivastava. Regulated proteolysis is a fundamental process used in the transmission of extracellular signals through the cell membrane into the cytosol. The role of regulated proteolysis in the communication of bacteria with the environment through modulation of bacterial signal transduction systems is reviewed by Wettstadt and Llamas.

Finally, in a mini-review, Jean-Pierre et al. present the latest advances in metabolic modeling, a computational method used to predict metabolic capabilities and interactions from individual microorganisms to complex polymicrobial communities in efforts to better understand microbial community function.

## Where Do We Go from Here?

The manuscripts that are compiled under the aegis of this Research Topic gave us an indication of the breadth and depth of research that has been carried out on prokaryotic communications in the post-genomic era—either intracellular, cell-cell, cell-host, or cell-environment. From the intricate intra- and intermolecular dance that characterizes the fundamental cellular processes of DNA replication, segregation, transcription, translation, and post-translational modifications to the complex but elegant interplay of the various components of the cellular regulatory networks, the wonders of the living prokaryotic cell never cease to amaze. The dictionary of the chemical language of bacterial cells continues to grow and we are constantly finding ways to exploit our knowledge of them to mitigate the spread of multidrug-resistant pathogens. The success of this Research Topic led us to launch a second volume where we hope to further explore the nooks, the crannies, the various alleyways, paths, avenues, and roads that lie ahead of us in our quest to understand how the most abundant living organisms on planet Earth talk.

## Author Contributions

All authors listed have made a substantial, direct and intellectual contribution to the work, and approved it for publication.

## Conflict of Interest

The authors declare that the research was conducted in the absence of any commercial or financial relationships that could be construed as a potential conflict of interest.
